# Organ specific regenerative markers in peri-organ adipose: kidney

**DOI:** 10.1186/1476-511X-10-171

**Published:** 2011-09-29

**Authors:** Joydeep Basu, Christopher W Genheimer, Namrata Sangha, Sarah F Quinlan, Kelly I Guthrie, Rusty Kelley, Roger M Ilagan, Deepak Jain, Timothy Bertram, John W Ludlow

**Affiliations:** 1 Bioprocess Research and Assay Development, Tengion Inc, 3929 Westpoint Blvd., Suite G, Winston-Salem, NC 27103, USA

**Keywords:** erythropoietin, EPO, adipose, kidney, chronic kidney disease, VEGF, WT1, regenerative medicine, tissue engineering, cell therapy

## Abstract

**Background:**

Therapeutically bioactive cell populations are currently understood to promote regenerative outcomes *in vivo *by leveraging mechanisms of action including secretion of growth factors, site specific engraftment and directed differentiation. Constitutive cellular populations undoubtedly participate in the regenerative process. Adipose tissue represents a source of therapeutically bioactive cell populations. The potential of these cells to participate in various aspects of the regenerative process has been demonstrated broadly. However, organ association of secretory and developmental markers to specific peri-organ adipose depots has not been investigated. To characterize this topographical association, we explored the potential of cells isolated from the stromal vascular fraction (SVF) of kidney sourced adipose to express key renal associated factors.

**Results:**

We report that renal adipose tissue is a novel reservoir for EPO expressing cells. Kidney sourced adipose stromal cells demonstrate hypoxia regulated expression of EPO and VEGF transcripts. Using iso-electric focusing, we demonstrate that kidney and non-kidney sourced adipose stromal cells present unique patterns of EPO post-translational modification, consistent with the idea that renal and non-renal sources are functionally distinct adipose depots. In addition, kidney sourced adipose stromal cells specifically express the key renal developmental transcription factor WT1.

**Conclusions:**

Taken together, these data are consistent with the notion that kidney sourced adipose stromal (KiSAS) cells may be primed to recreate a regenerative micro-environment within the kidney. These findings open the possibility of isolating solid-organ associated adipose derived cell populations for therapeutic applications in organ-specific regenerative medicine products.

## Background

Adipose is recognized as an endocrine organ with significant metabolic bioactivity. Adipose tissue is composed of adipocytes, vascular endothelial cells, pericytes, fibroblasts, macrophages, stem cells and progenitors with MSC-like bioactivity and smooth muscle-like cells [1-4]. Of these, MSC-like and smooth muscle-like cell populations are currently under active development for application in tissue engineering and regenerative medicine [5]. At a higher level, adipose tissue may be classified as white or brown based on the preponderance of white or brown adipocytes. White adipocytes represent the principal lipid storage vehicle within adipose tissue, whereas brown adipocytes are responsible for mediating lipid metabolism and are therefore correspondingly enriched in mitochondria. Adipose tissue may be found distributed broadly throughout the body as distinctive, region specific depots. The principal depots for white adipose tissue (WAT) are abdominal subcutaneous and visceral adipose tissue (SAT and VAT). VAT may itself be further subdivided into omental, mesenteric, retroperitoneal, gonadal and pericardial depots [6,7]. Adipose depots are characterized by unique patterns of structural organization, transcriptomic, proteomic and secretomic expression profiles and biological function. For example, secretomes generated by visceral, subcutaneous and gonadal adipose depots are specific to source [8]. Furthermore, significant functional differences between subcutaneous, epididymal and mesenteric adiposes have been observed through transcriptomic and lipidomic analysis of transgenic mice with humanized lipoprotein profiles [9]. Finally, the multi-lineage differentiation potential of adipose-derived stromal cells with MSC-like bioactivity has been shown to be dependant on the depot of origin [10,11]. These systemic observations notwithstanding, analysis and characterization of transcriptomic, proteomic and functional differences between adiposes associated with individual organs remains to be investigated. More specifically, understanding the variation in regenerative potentials presented by stromal cells derived from differently sourced solid organ associated adiposes may significantly impact the development of tissue engineering and regenerative medicine (TE/RM) products targeted to those organs. As a follow-up to our recently reported neo-kidney augment work [12], we have focused in the current study on evaluation of key functional criteria discriminating stromal cells derived from kidney and non-kidney sourced adiposes through analysis of established regenerative and developmental markers associated with kidney: erythropoietin (EPO), VEGF and WT1. We demonstrate for the first time that renal adipose tissue presents depot specific expression of EPO, and that stromal cell populations derived from kidney and non-kidney sourced adiposes express EPO and VEGF in a hypoxia-regulated manner. Furthermore, we show that expression of the key nephrogenic transcription factor WT1 is specific to kidney adipose sourced stromal cells, and that niche specific adipose depots within kidney may be defined by distinctive WT1 transcriptional splice variants. Taken together, these data extend the concept of functionally unique, location-specific adipose depots from the systemic to the organ-level, and establish a foundation for application of kidney sourced adipose as an alternate cell source for tissue engineering and regenerative therapies of the kidney.

## Results

### EPO is expressed by multiple cell sources

**Figure 1:**
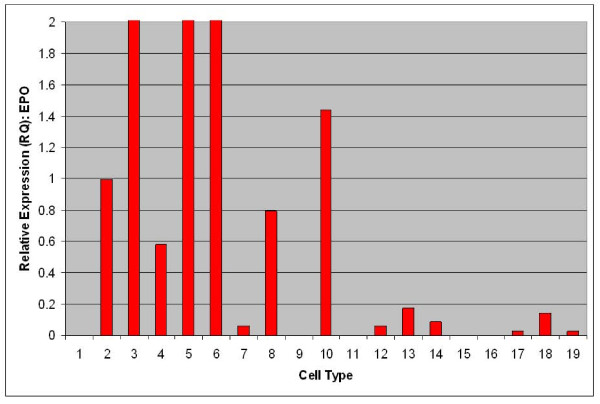
**EPO is expressed by multiple cell types as determined by TaqMan qRTPCR comparative analysis of the indicated human cell and tissue sources**. Human primary renal cells were used as calibrator and have a designated RQ value of 1.0. Samples are as follows: H_2_O as a negative control (1); human primary renal cells (2); whole fetal liver (3); CD34+ enriched fetal liver cells (4); hepatocytes (5); keratinocytes at passage 4 (6); human dermal microvascular endothelial cells (7); human epidermal keratinocytes (8); peripheral blood derived mononuclear cells, CD34+, GCSF mobilized (9); peripheral blood derived mononuclear cells, CD34+, normal (10); adipose (lipoaspirate) stromal cells at passage 0 (11); passage 1 (12); passage 2 (13); passage 4 (14); adipose (subcutaneous) stromal cells at passage 0 (15); passage 1 (16); passage 2 (17); passage 3 (18); and passage 4 (19).

**Figure 2:**
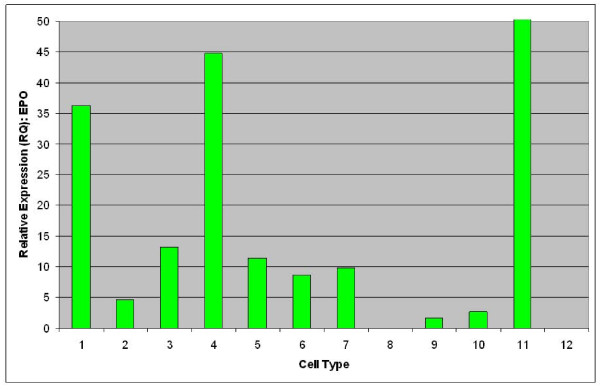
**Expression of EPO from kidney and non-kidney sourced adipose stromal cells is comparable to primary renal cells as determined by TaqMan qRT-PCR comparative analysis**. Samples are as follows: Primary renal cells (1 and 2); primary renal cells exposed to hypoxic conditions (3); adipose lipoaspirate (4) and subcutaneous (5) stromal cells at passage 0; adipose lipoaspirate (6) and subcutaneous (7) stromal cells at passage 1; major calyx derived kidney sourced adipose stromal cells at passage 1 (8); renal pedicle derived kidney sourced adipose stromal cells at passage 1 (9) and passage 3 (10); fetal hepatocytes used as a positive control (11); H_2_O used as a negative control (12).

To evaluate expression of EPO mRNA from adipose-derived stromal cells relative to established sources of EPO, TaqMan quantitative RT-PCR was performed [13]. As shown in Figure 1, strong expression of EPO is observable from renal primary cells (cell type 2), fetal liver (cell type 3), adult hepatocytes (cell type 5), keratinocytes (cell types 6 and 8) and non-mobilized CD34+ PBMNC (cell type 10). Mobilization of CD34+ PBMNC with GCSF was observed to lead to silencing of EPO mRNA expression (cell type 9). Relatively lower levels of EPO mRNA were observed from multiple samples of non-kidney sourced adipose stromal cells such as CD34+ enriched fetal liver cells (cell type 4) as well as passaged lipoaspirate (cell types 11-14) and subcutaneous (cell types 15-10) adipose stromal cells. EPO expression was also evaluated from kidney and non-kidney sourced adipose stromal cells directly against renal primary cells. As shown in Figure 2, expression of EPO mRNA from both kidney and non-kidney sourced adipose stromal cells (cell types 4-7, 9, 10) is comparable to that observed from primary kidney cells (cell types 1-3).

### Expression of EPO mRNA from kidney and non-kidney sourced adipose stromal cells is regulated by hypoxia

**Figure 3:**
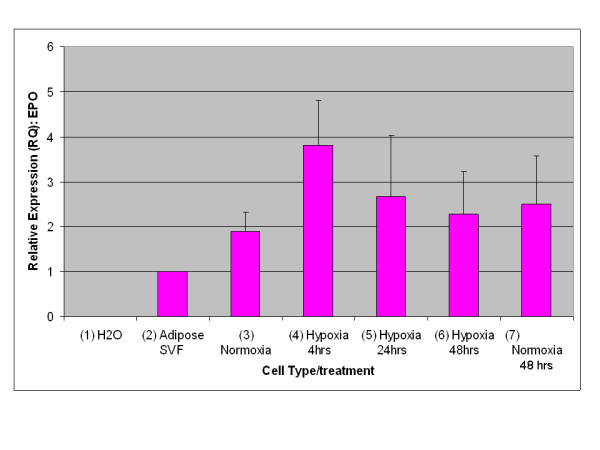
**Regulated expression of EPO from rat visceral adipose stromal cells as determined by TaqMan qRT-PCR analysis**. EPO expression is up-regulated in response to hypoxia within 4 hours, and returns towards baseline within 48 hours of transfer to normoxia (*n *= 3).

**Figure 4:**
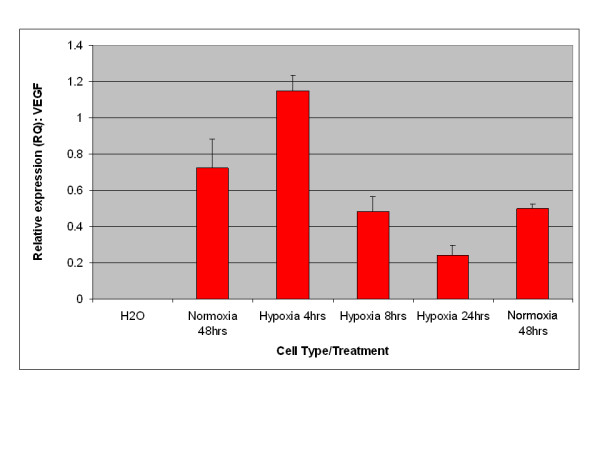
**Regulated expression of VEGF from rat visceral adipose stromal cells as determined by TaqMan qRT-PCR analysis**. VEGF expression is up-regulated in response to hypoxia within 4 hours, and returns towards baseline within 48 hours of transfer to normoxia (*n *= 3).

**Figure 5:**
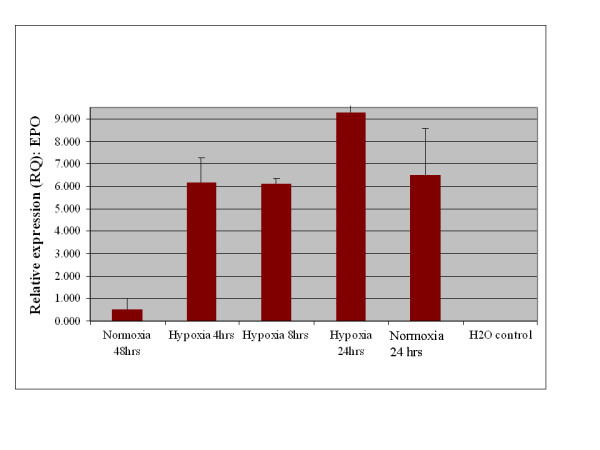
**Regulated expression of EPO from human renal pedicle adipose stromal cells as determined by TaqMan qRT-PCR analysis**. EPO expression is up-regulated in response to hypoxia within 4 hours, and returns towards baseline for renal pedicle sourced adipose (*n *= 3).

**Figure 6:**
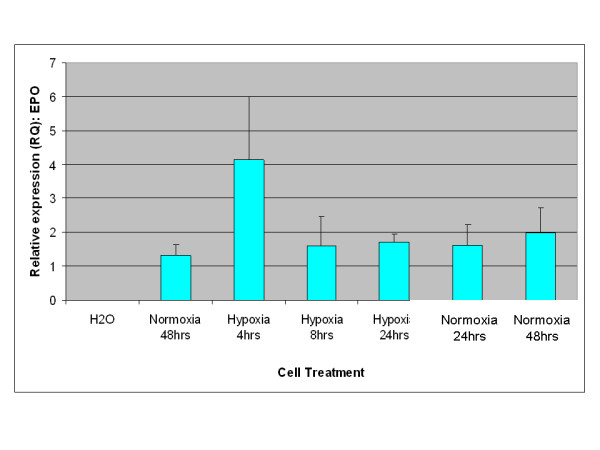
**Regulated expression of EPO from human major calyx adipose stromal cells as determined by TaqMan qRT-PCR analysis**. EPO expression is up-regulated in response to hypoxia within 4 hours, and returns towards baseline within 48 hours of transfer to normoxia (*n *= 3) for major calyx sourced adipose.

**Figure 7:**
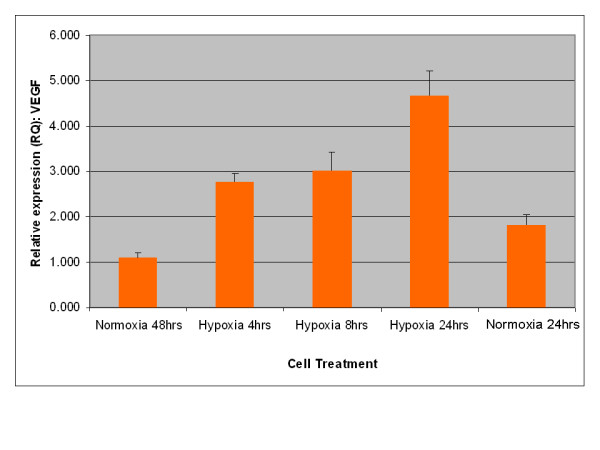
**Regulated expression of VEGF from human renal pedicle adipose stromal cells as determined by TaqMan qRT-PCR analysis**. VEGF expression is up-regulated in response to hypoxia within 4 hours, and returns towards baseline within 48 hours of transfer to normoxia (*n *= 3).

**Figure 8:**
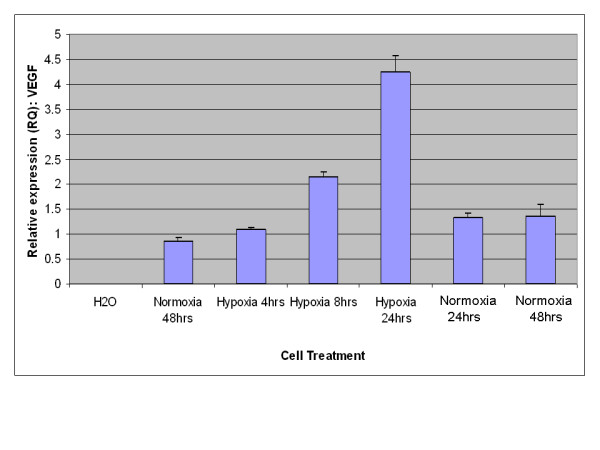
**Regulated expression of VEGF from human major calyx adipose stromal cells as determined by TaqMan qRT-PCR analysis**. VEGF expression is up-regulated in response to hypoxia within 4 hours, and returns towards baseline within 48 hours of transfer to normoxia (*n *= 3).

A key characteristic of EPO expression from kidney is regulation by environmental oxygen [14,15]. Based on this criterion, an experiment was performed to establish whether expression of EPO from adipose stromal cells was physiologically relevant by quantifying the production of EPO mRNA under conditions of high and low environmental oxygen. Initial studies focused on rodents, as the rat is the small animal model of choice for the investigation of CKD [16]. As shown in Figure 3, rat visceral adipose stromal cells respond to hypoxia by an up-regulation of the expression of EPO mRNA within 4 hours of treatment. Expression of VEGF mRNA from rat visceral adipose stromal cells is also tightly controlled by hypoxia, showing up-regulation within 4 hours of treatment and subsequent down-regulation within 48 hours of return to normoxia (Figure 4). This result is reflected by human KiSAS cells. As shown in Figures 5, 6, 7, 8, hypoxia induced up-regulation of EPO and VEGF mRNA expression was observed from human renal pedicle and major calyx sourced adipose stromal cells.

### Expression of EPO protein is comparable between human KiSAS cells and human renal primary cells

**Figure 9:**
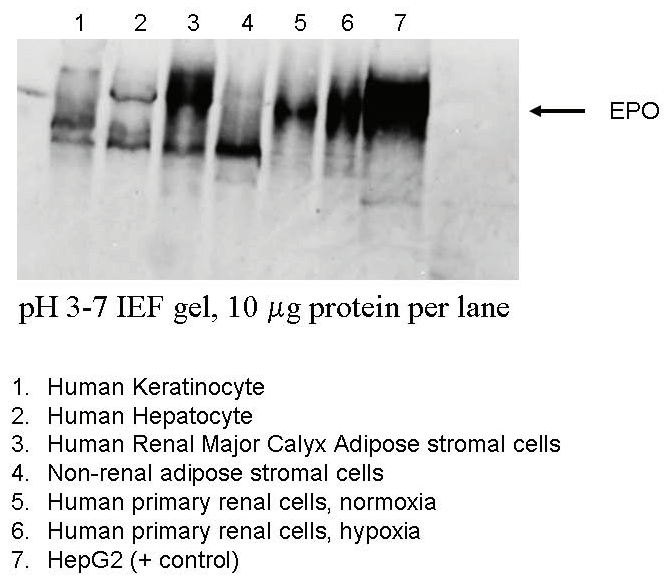
**Expression of EPO from human kidney and non-kidney sourced adipose stromal cells is comparable with primary renal cells, hepatocytes and keratinocytes**. Kidney and non-kidney sourced adipose stromal cells express distinct isoforms of EPO distinguishable though isoelectric focusing gel electrophoresis and western blotting. Samples are as follows: human keratinocytes (1), hepatocytes (2), renal- (3) non-renal- (4) adipose stromal- cells and primary renal-cells all under normoxia (5), primary renal cells under hypoxia (6) and HepG2 cells as a positive control (7). Blot was probed with anti-EPO monoclonal antibody for Western analysis. Comparison of lanes 5 and 6 shows clear up-regulation of EPO protein expression in response to hypoxia from primary renal cell isolates. All lanes were normalized by total mass of protein (10 μg).

Although expression levels of EPO mRNA in adipose stromal cells are significantly lower relative to primary renal cells, quantitation of EPO mRNA is secondary from a functional or bio-therapeutic perspective to evaluation of EPO protein expression between differently sourced adiposes. We therefore investigated expression of EPO protein from adipose sourced stromal cells using iso-electric focusing gels (IEF). IEF technology has been used to discriminate between multiple, differently modified isoforms of EPO with high resolution [17]. Figure 9 demonstrates that EPO protein is expressed from human KiSAS cells (lane 3) at levels directly comparable to that observed from established cell sources of EPO, including keratinocytes (lane 1), hepatocytes (lane 2), and primary kidney cells (lanes 5, 6) [18,19]. EPO is also expressed by visceral (non-renal) adipose stromal cells (lane 4), but at significantly reduced levels. Kidney and non-kidney sourced adipose stromal cells show distinctive patterns of post-translational EPO modification resulting in unique migration profiles on IEF gels, as can be seen by comparing lane 3 with lane 4. In addition, EPO from kidney or non-kidney sourced adipose stromal cell sources is further distinguishable from EPO expressed by primary renal cells on the basis of iso-electric point (compare lanes 3 and 4 with lanes 5 and 6). Finally, EPO isoforms expressed by human keratinocytes and hepatocytes (lanes 1 and 2) are distinguishable from all other cell sources.

**Figure 10:**
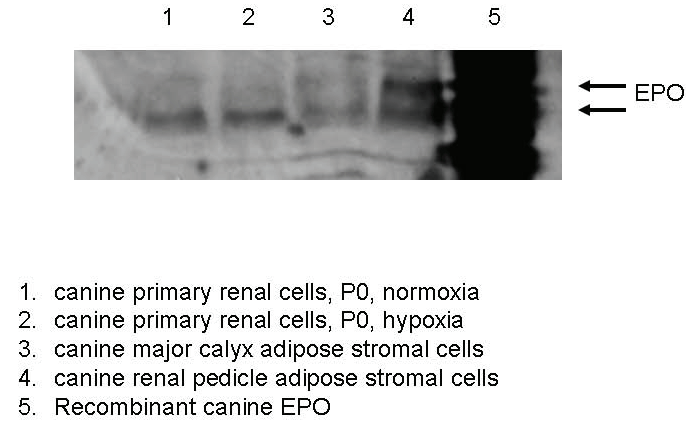
**Expression of EPO from canine KiSAS cells is comparable to canine primary renal cells**. Samples are as follows: canine primary renal cells under normoxia (1) and hypoxia (2), major calyx-derived adipose stromal cells (3), renal pedicle derived-adipose stromal cells (4) and recombinant canine EPO as a positive control (5). Blot was probed with anti-EPO monoclonal antibody for Western analysis. All lanes were normalized by total mass of protein (10 μg)

As dog is the large animal model of choice for evaluation of renal cell therapies [20], we examined expression of EPO protein in canine kidney sourced adipose stromal cells and primary renal cells using IEF gels. Figure 10 demonstrates that canine KiSAS cells sourced from either major calyx adipose (lane 3) or renal pedicle adipose (lane 4) express EPO protein at levels comparable to that observed from canine primary renal cells (lanes 1 and 2). Additionally, as is the case for human adipose, EPO expressed from stromal cells sourced from either the renal pedicle or major calyx have unique IEF signatures that discriminate canine adipose sourced EPO from that expressed by canine primary renal cells. Taken together, these data establish that KiSAS cells express EPO protein at levels comparable to that seen from established cellular sources of EPO including renal cells, hepatocytes and keratinocytes and confirm that differently sourced EPO may be identified by iso-electric point profiling [17-19].

### Kidney sourced adipose tissue is an organ-specific reservoir for EPO

**Figure 11:**
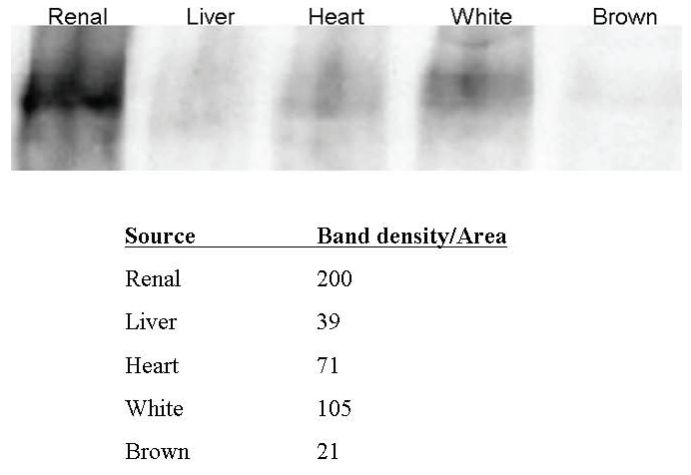
**Adipose tissue is a reservoir for EPO expressing cells distinguishable though isoelectric focusing gel electrophoresis and western blotting**. White and brown adipose are derived from visceral depot. All lanes normalized by mass of protein to 10 μg. Table shows quantitative densitometric analysis of EPO expression expressed as band intensity per unit gel area.

To establish if expression of EPO from adipose stromal cell populations reflects a physiologically relevant EPO reservoir *in vivo*, or is simply an artifact associated with cell culturing, we examined expression of EPO protein from differently sourced rat whole adipose *tissue *by IEF. As shown in Figure 11 where individual lanes are normalized by mass protein loaded, robust expression of EPO is specifically associated with kidney sourced adipose tissue. Although EPO is detectable from non-kidney organ sources of adipose tissue such as liver and heart, expression is 5-fold higher in kidney sourced adipose over liver sourced adipose and 2.8-fold higher in kidney sourced adipose over heart sourced adipose. Interestingly, comparison of white and brown adiposes sourced from visceral adipose depots shows EPO expression is 5-fold higher in white adipose over brown adipose.

### Expression of the developmental transcription factor WT1 distinguishes kidney from non-kidney sourced adipose stromal cells

**Figure 12:**
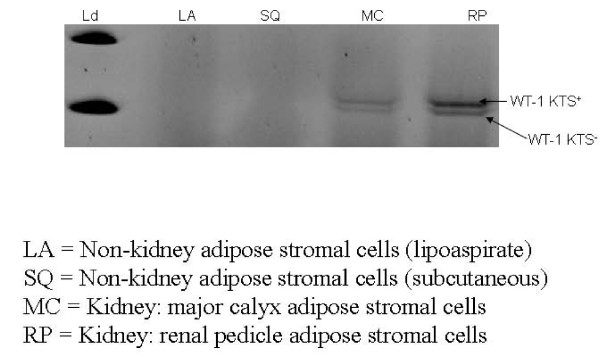
**Semi-quantitative RT-PCR analysis of WT1 splice variants (KTS+/KTS-) in human kidney and non-kidney sourced adipose stromal cells**. All lanes were normalized by total mass of cDNA: molecular weight ladder used for sizing (1), lipoaspirate stromal cells (Lane 2), subcutaneous adipose stromal cells (Lane 3), major calyx adipose stromal cells (Lane 4), renal pedicle stromal cells (Lane 5). Expression of WT1 is only detectable from renal adipose. Note that the ratio of KTS+/KTS- splice variants differs between major calyx and renal pedicle derived cell sources derived from the same donor.

**Figure 13:**
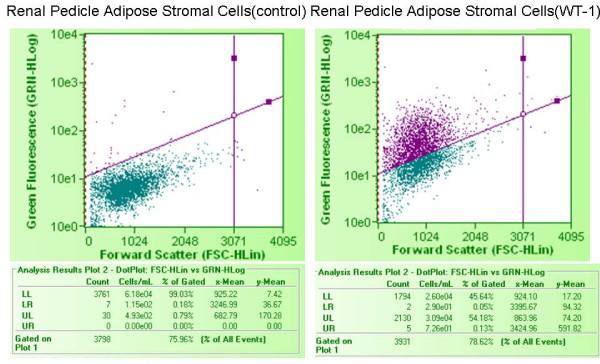
**FACs analysis of the distribution of WT1+ cells in renal pedicle associated adipose stromal cells**. 45.6% of renal pedicle adipose stromal cell population was WT1+.

**Figure 14:**
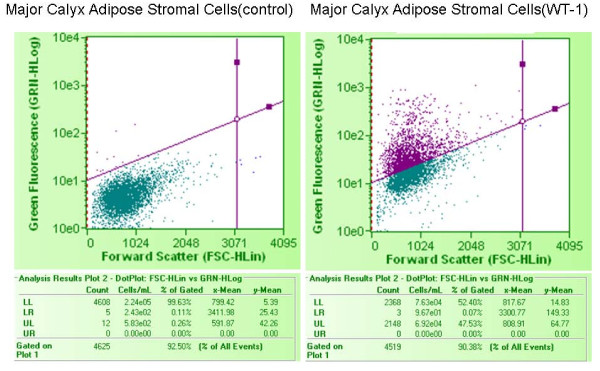
**FACs analysis of the distribution of WT1+ cells in major calyx associated adipose stromal cells**. 52.4% of major calyx adipose stromal cell population was WT1+.

**Figure 15:**
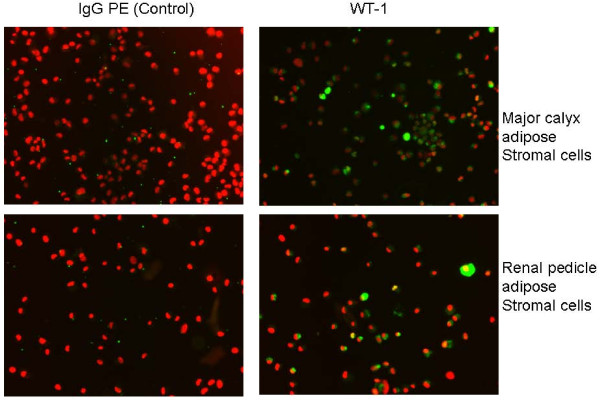
**Immuno-fluorescence analysis of the distribution of WT1+ cells in major calyx adipose stromal cells and renal pedicle adipose stromal cells**. Note that localization of WT1 expression is cytoplasmic and nuclear. WT1 (green), DNA (red).

We reasoned that renal markers other than EPO might be associated with organ specific expression patterns within differently sourced adiposes. WT1 is a key zinc finger transcription factor broadly involved in organogenesis. WT1 acts to modulate the earliest stages of nephrogenesis, and may serve as a marker for regeneration [21-23]. We used primers specific to the KTS+ and KTS- transcriptional splice variants of WT1 [24] to investigate the expression of WT1 within kidney and non-kidney sourced adipose stromal cells. As shown in Figure 12, expression of WT1 mRNA is specific to kidney sourced adipose stromal cells (lanes MC and RP), with no expression being observed from visceral adipose stromal cells (lanes LA and SQ). Interestingly, the ratio of these two splice variants differs between stromal cells sourced from major calyx adipose (lane MC) or renal pedicle adipose (lane RP) derived from the same donor. We extended the WT1 RT-PCR analysis by evaluating expression of WT1 protein in KiSAS cells through FACs and immuno-fluorescence approaches. Figures 13, 14 show that both renal pedicle and major calyx sourced adipose stromal cells have a WT1+ population, ranging from approximately 45% (renal pedicle) to 52% (major calyx) of the population. Expression of WT1 is both nuclear and cytoplasmic, (Figure 15), as has been previously reported [25].

## Discussion

Cell therapy strategies for the treatment of CKD have typically focused on application of mesenchymal stem cells (MSC) (renal or bone-marrow derived) or embryonic and fetal progenitors. Evidence for therapeutic efficacy remains mixed, with little or no demonstration of localized engraftment and differentiation towards an endogenous renal cell population [26,27]. Current thinking regarding the therapeutic mechanism of action of MSC and other related progenitor populations has emphasized their trophic function as cellular vectors for secreting large quantities of cytokines and growth factors into the extracellular milieu, thereby facilitating the re-creation of a regenerative micro-environment [28]. To this end, cells with MSC-like bioactivity may be sourced from multiple organ systems including adipose [1,2]. However, differently sourced MSC-like cells may not be functionally equivalent. For example, numerous functional and phenotypic differences have been documented between MSC sourced from either adipose or bone marrow [5,12]. MSC bioactivity *within *a given organ source may also be variable. Within adipose for instance, MSC bioactivity is contingent on the specific depots from which the adipose was sourced [10,11]. Therefore, MSC bioactivity may be regarded as a functional indicator discriminating differently sourced adiposes. Additional studies have also identified depot specific functional features of differently sourced adiposes, such as regional differences in micro-RNA expression and androgen concentration between human omental and subcutaneous adiposes [29,30]. Finally, disparity in the functional properties of white and brown adipose tissue is well described [31]. These observations led us to further evaluate organ specific functional differences between differently sourced adiposes. For the current study, we focused on organ specific features associated with kidney sourced adipose, beginning with analysis of the kidney associated regenerative growth factor EPO.

The regulated expression of EPO is a key mechanistic pathway for the control of erythropoiesis. EPO, a 34 kDa glycoprotein principally expressed from fetal liver and adult kidney, is up-regulated through the O_2_-dependant modulation of HIF-1α/2α activity. Binding of these transcription factors to the appropriate regulatory regions of the EPO genomic locus triggers concomitant synthesis of EPO mRNA [14,15]. To date, expression of EPO has been detected in the kidney, liver, brain, spleen, lung and testis [32]. Within the kidney, the peri-tubular interstitial fibroblasts of the cortex and medulla have been implicated as the site of EPO production [33,34], and an EPO expressing primary renal cell population has recently been described [35]. Secretion of EPO from the kidney is required for regulation of erythropoeisis and to avoid severe anemia secondary to CKD [36]. Attempts to directly address CKD-associated anemia through treatment with recombinant EPO have generated mixed results [37,38], leading to efforts to develop gene and cell therapy based approaches for the targeted delivery of EPO in a physiologically relevant manner [35,39,40]. From a broader perspective, the secretion of critical trophic factors such as EPO and VEGF may be the principal mechanism responsible for observed therapeutic efficacy of MSC in chronic and acute injury models [28]. The angiogenic activity of VEGF may additionally serve an anti-fibrotic function, acting to further retard deterioration of the renal environment [41]. EPO may additionally function to enhance cell survival [42,43].

In the current report, we show that kidney sourced adipose represents a hitherto unidentified reservoir of EPO producing cells. As with other previously documented sources of EPO [14,15,32,33], expression of EPO mRNA from kidney sourced adipose stromal cells is regulated by environmental oxygen. Observed down-regulation of EPO mRNA expression during the hypoxia-normoxia transition is not as tightly regulated as that shown by VEGF mRNA (Figures 5, 6, 7, 8), despite both VEGF and EPO mRNAs being responsive to hypoxia through the same HIF-1α/2α mediated regulatory pathways [15]. This apparently less stringent regulatory control of EPO mRNA is likely due to the significantly lower relative expression levels of EPO mRNA compared to VEGF mRNA [44]. That expression of EPO is not an artifact generated from cell culture is demonstrated by the observation that kidney sourced adipose tissue is a reservoir for EPO expression (Figure 11). Interestingly, Figure 11 also shows that although EPO expression from non-kidney sourced adipose is significantly lower relative to kidney sourced adipose, EPO is nevertheless a specific marker within visceral sourced adipose discriminating white from brown adipocytes. As shown in Figure 9, expression of EPO from KiSAS cells is equivalent to that observed from primary renal cell populations currently under development for cell therapy of anemias secondary to CKD [35], suggesting that kidney sourced adipose may represent an alternate cell source for cellular vectors for EPO delivery.

Variation in the iso-electric isotype signature of EPO within urine and corresponding serum has been previously documented [45]. Iso-electric isotype profiling has been used to discriminate recombinant from native EPO in urine of athletes suspected of illicit self-medication [19]. Similarly, IEF gel electrophoresis analysis of stromal cell populations sourced from kidney and non-kidney depots shows that such cell types may be functionally distinguished by unique differences in the pattern of post-translational modification of EPO (Figure 9). Furthermore, additional iso-electric point isotypes of EPO are observable within differently sourced adiposes originating from the same organ, as demonstrated by the distinctive IEF signatures of EPO expressed by major calyx or renal pedicle-sourced adipose stromal cells (Figure 10). This data lends credence to the notion that major calyx and renal pedicle represent distinct adipose depots within kidney.

Our observations on the differential post-translational modification patterns of EPO expressed by kidney and non-kidney sourced adipose stromal cells led us to investigate the expression status of additional key renal markers that could potentially serve to further distinguish between renal and non-renal adipose depots. To this end, WT1 is a zinc finger transcription factor broadly involved in organogenesis. WT1 acts to modulate the earliest stages of nephrogenesis, and may serve as a marker for regeneration [21-23]. Expression of WT1 mRNA is specific to kidney sourced adipose stromal cells (Figure 12). WT1 mRNA is not detected from viscerally sourced adipose. Transcriptional regulation of WT1 is complex and multiple splice variants with distinctive biological functions have been characterized. The KTS+ and KTS- variants of WT1 differ in the presence or absence of 3 amino acids (KTS) between zinc fingers 3 and 4. The ratio of KTS+/KTS- WT1 splice variants is reported to be constant across multiple tissues and the manipulation of this balance can trigger severe developmental anomalies [24]. The observation that kidney sourced adipose stromal cells (but *not *non-kidney sourced adipose stromal cells) specifically express WT1 is consistent with observed differences in expression levels and in post-translational modification of EPO between kidney and non-kidney sourced adipose stromal cells (Figure 9). Observed differences in the ratio of KTS+/KTS- WT1 splice variants within differently sourced adipose stromal cells derived from the same organ of the same individual is remarkable; such disparity is contrary to previous reports [24], but is consistent with organ and niche specific variability in EPO IEF signatures documented herein, providing further confirmation of depot specific functionalities within adipose. Interestingly, only approximately 50% of the stromal cell population derived from either major calyx or renal pedicle depots is WT1+ (Figure 13, 14, 15), suggesting that two functionally distinct cellular sub-populations may be present within these adiposes. We are currently separating these WT1+ and WT1- sub-populations to further determine the biological significance of this observation.

## Conclusions

In conclusion, we show in this report that adipose tissue is a novel source of EPO. We demonstrate that expression of EPO and VEGF mRNAs from adipose (kidney and non-kidney sources) is regulated by environmental oxygen and is directly comparable to that observed from primary renal cells or other established sources of EPO. Renal and non-renal sources of adipose have unique functional properties manifested through differences in the level of expression and post-translational modification of EPO. These data suggest that renal and non-renal adipose represent fundamentally distinct adipose depots. To this end, EPO protein expressed by kidney and non-kidney sourced adipose stromal cells is distinguishable based on distinctive migration patterns through IEF gels, a consequence of differences in the post-translational modification of EPO between the two cell types. Furthermore, KiSAS cells recapitulate several additional aspects of the functional phenotype of primary renal cells including expression of the key nephrogenic transcription factor WT1, leading us to speculate that renal adipose may be amenable towards acquisition of tubular functionality. In this regard, we have observed induction of established tubular markers within primary cultures of KiSAS cells in response to modulation with known morphogenic agents such as retinoic acid (our unpublished observations). Renal adipose may represent a potentially ideal alternate cell source for treatment of chronic anemia secondary to CKD, since renal adipose can be isolated in much larger quantities than renal primary cells and, as is the case for adipose associated with tubular organs such as bladder, may be unaffected by the occurrence of metastasis in renal cancer patients [46,47].

## Methods

### Isolation of renal and non-renal adipose-derived cells

Human non-renal adipose was obtained either subcutaneously or through visceral liposuction (Zen-Bio, http://www.zen-bio.com). Renal adipose from the renal pedicle was dissected away from human kidney (normal adult) donated through National Disease Research Institute (NDRI) in compliance with all NIH guidelines governing the use of human tissues for research purposes. Renal adipose from the major calyx was obtained by bisection of whole human kidney and dissection of calyx fat away from the medulla. Canine kidneys for isolation of canine renal adipose were obtained from the University of North Carolina at Chapel Hill (gift of Dr. Timothy Nichols). Rat kidneys for isolation of rat visceral and organ associated adiposes were sourced from male Lewis rats obtained from Charles River Labs. Regardless of species or tissue origin, all adipose samples were processed as follows: Adipose was extensively washed with PBS/0.1% gentamicin (Invitrogen-Gibco) and digested for up to 1 hour with 0.3% collagenase I (Worthington), 1% BSA in DMEM-HG (Invitrogen-Gibco) at 37°C. Samples were centrifuged at 600 *g *for 20 minutes and the adipocytic supernatant aspirated away. The remaining stromal vascular fraction was re-suspended in α-MEM/10% FBS (Invitrogen-Gibco) and placed in a tissue culture incubator for 24-48 hours. Non-adherent cell populations were removed by washing 3× with PBS. For experiments involving hypoxic inductions, cells were maintained in an O_2_-enriched (2%) incubator for the time periods indicated. VEGF mRNA expression was used as a control to confirm integrity of hypoxic regulation pathways.

### Non-Adipose Cells

Renal primary kidney cells from rat or human kidneys were isolated as previously described (12). Human peripheral blood derived mononuclear cells were isolated as described (13). CD34+ GCSF mobilized/non-mobilized peripheral blood mononuclear cell cDNA was purchased from AllCells LLC. Fetal and adult hepatocyte cDNA and keratinocyte cDNA were purchased from ScienCell Research Laboratories.

### TaqMan qRT-PCR

RNA was purified from cell samples using the RNeasy Plus Mini Kit (Qiagen) according to the manufacturer's instructions. cDNA was generated from the entire final volume of RNA using the SuperScript VILO cDNA Synthesis Kit (Invitrogen-Gibco) according to the manufacturers' instructions. Following cDNA synthesis, each sample was diluted 1:10 and used directly to set up qRT-PCR as follows: 10 μl master mix (2×), 1 μl primer/probe, 9 μl cDNA. The following TaqMan primer/probe sets were procured from Applied Biosystems: rat EPO: Rn01481376_m1, Rat VEGF-A: Rn00582935, human EPO: Hs00171267_m1, human VEGF-A: Hs00900058_ml, human WT-1: Hs01103754_m1. All TaqMan reactions were carried out in an ABI 7300 real time thermal cycler using default cycling parameters. Analysis of PCR data was performed using the method of Relative Quantitation (RQ) by comparative Ct.

### Iso-electric focusing gel analysis of EPO

Up to 1 × 10^6 ^cells or up to 30 mg total adipose tissue was lysed in protein lysis buffer (50 mM Tris pH 8.0; 150 mM NaCl; 0.5% NP40 and protease inhibitors, Roche). 10 μg of protein lysate was loaded onto a pH 3-7 iso-electric focusing (IEF) gel (Invitrogen-Gibco) and run out as recommended by the manufacturer. IEF gels were transferred to nitrocellulose using the iBlot transfer system (Invitrogen-Gibco) and probed overnight with MAB 2871 anti-hEPO monoclonal antibody (R&D Systems) at 1/500 in TBST (Tris-buffered saline, pH 7.0, 0.1% Tween-20)/2% milk. Secondary antibody was horse anti-mouse IgG/HRP conjugate (Vector Labs) at 1/60000 in TBST/2% milk.

### FACs and immuno-fluorescence

Cells (0.5 × 10^6^- 1 × 10^6^) were fixed in 2% para-formaldehyde and F_c _receptors blocked to prevent non-specific binding. Cells were permeabilized by incubation in PBS/0.2% Triton X-100/10% horse serum for 30 minutes. Cells were then incubated with a directly conjugated antibody against human WT1 (Santa Cruz) as recommended by the manufacturer. Subsequent to final washing (PBS, 0.2% Triton X-100), antigen detection was performed utilizing the Guava EasyCyte Mini Express Assay system using the appropriate fluorescent channel. A minimum of 5, 000-10, 000 events were acquired from each sample. For immuno-fluorescence analysis, the labeled cell suspension was centrifuged directly onto a poly-L-lysine coated slide (Electron Microscopy Sciences) using a cyto-centrifugation system (Viescor) at 1, 500 rpm for 5 minutes. Cells were counterstained with DAPI containing mounting medium (Vector Labs) and viewed with a Leica DMI 4000 B fluorescence microscope.

## List of abbreviations

CKD: Chronic Kidney Disease; EPO: Erythropoietin; IEF: Iso-Electric Focusing; MSC: Mesenchymal Stem Cell; KiSAS: Kidney Sourced Adipose Stromal cells; PBMNC: Peripheral Blood Mononuclear Cells; SAT: Subcutaneous Adipose Tissue; SVF: Stromal Vascular Fraction; TE/RM: Tissue Engineering/Regenerative Medicine; VEGF: Vascular Endothelial Growth Factor; VAT: Visceral Adipose Tissue; WAT: White Adipose Tissue; WT1: Wilm's Tumor Protein 1.

## Competing interests

The authors declare that they have no competing interests.

## Authors' contributions

JB and JWL conceived the experiments and wrote the manuscript. JB, CG, NS, SFQ, KIG performed the experiments. RK, RIM, DJ and TB provided critical oversight and assisted with interpretation of experiments. All authors read and approved the final manuscript.
